# Maternal lipid profile in pregnancy and embryonic size: a population-based prospective cohort study

**DOI:** 10.1186/s12884-022-04647-6

**Published:** 2022-04-18

**Authors:** Dionne V. Gootjes, Anke G. Posthumus, Deveney F. Wols, Yolanda B. de Rijke, Jeanine E. Roeters Van Lennep, Eric A. P. Steegers

**Affiliations:** 1 Department of Obstetrics and Gynecology, Division of Obstetrics and Fetal Medicine, Erasmus MC University Medical Centre, Rotterdam, The Netherlands; 2 Generation R Study Group, Erasmus MC University Medical Centre, Rotterdam, The Netherlands; 3 Department of Clinical Chemistry, Erasmus Medical Center Rotterdam, Erasmus MC University Medical Centre, Rotterdam, The Netherlands; 4 Department of Internal Medicine, Erasmus Medical Center, Erasmus MC University Medical Centre, Rotterdam, The Netherlands

**Keywords:** Pregnancy, Cholesterol, Low-density lipoprotein (LDL-c), High-density lipoprotein (HDL-c), Triglycerides, Intrauterine development, Fetal growth, Early pregnancy

## Abstract

**Background:**

Lipids are crucial for fetal growth and development. Maternal lipid concentrations are associated with fetal growth in the second and third trimester of pregnancy and with birth outcomes. However, it is unknown if this association starts early in pregnancy or arises later during fetal development. The aim of this study was to investigate the association between the maternal lipid profile in early pregnancy and embryonic size.

**Methods:**

We included 1474 women from the Generation R Study, a population based prospective birth cohort. Both embryonic size and the maternal lipid profile were measured between 10 weeks + 1 day and 13 weeks + 6 days gestational age. The maternal lipid profile was defined as total cholesterol, triglycerides (TG), high-density lipoprotein cholesterol (HDL-c), low-density lipoprotein cholesterol (LDL-c), remnant cholesterol, non-high-density (non-HDL-c) lipoprotein cholesterol concentrations and the triglycerides/high-density lipoprotein (TG/HDL-c) ratio. Additionally, maternal glucose concentrations were assessed. Embryonic size was assessed using crown-rump length (CRL) measurements. Associations were studied with linear regression models, adjusted for confounding factors: maternal age, pre-pregnancy body mass index (BMI), parity, educational level, ethnicity, smoking and folic acid supplement use.

**Results:**

Triglycerides and remnant cholesterol concentrations are positively associated with embryonic size (fully adjusted models, 0.17 SDS CRL: 95% CI 0.03; 0.30, and 0.17 SDS: 95% CI 0.04; 0.31 per 1 MoM increase, respectively). These associations were not present in women with normal weight (triglycerides and remnant cholesterol: fully adjusted model, 0.44 SDS: 95% CI 0.15; 0.72). Associations between maternal lipid concentrations and embryonic size were not attenuated after adjustment for glucose concentrations. Total cholesterol, HDL-c, LDL-c, non-HDL-c concentrations and the TG/HDL-c ratio were not associated with embryonic size.

**Conclusions:**

Higher triglycerides and remnant cholesterol concentrations in early pregnancy are associated with increased embryonic size, most notably in overweight women.

**Trial registration:**

The study protocol has been approved by the Medical Ethics Committee of the Erasmus University Medical Centre (Erasmus MC), Rotterdam (MEC-2007-413). Written informed consent was obtained from all participants.

**Supplementary Information:**

The online version contains supplementary material available at 10.1186/s12884-022-04647-6.

## Background

In pregnancy, lipids are crucial for the developing fetus and to maintain placental function [1]. Lipids are fatty substances that are either absorbed from food or synthesized by the liver, and comprise of cholesterol, triglycerides and lipoproteins. Cholesterol is crucial to provide structural integrity to the cell membrane [2, 3].

Triglycerides are nonpolar lipid molecules associated with various lipoproteins that primarily store energy in adipocytes and muscle cells. Lipoproteins are structures that possess surface proteins that are cofactors and ligands for lipid-processing enzymes. They are classified by their size and density as either low-density lipoprotein cholesterol (LDL-c) and high-density lipoprotein cholesterol (HDL-c) [4]. Low-density lipoproteins transport cholesterol, the main substrate for progesterone synthesis, and thereby support the maintenance of a pregnancy [5, 6]. By receptor-mediated endocytosis, maternal LDL can be taken up into the syncytiotrophoblast [7]. After uptake, LDL-derived triglycerides and cholesterol are hydrolyzed by placental lipases and contribute to the intracellular fatty acid pool. HDL binds to receptors on the syncytiotrophoblast surface and is subsequently hydrolyzed by extracellular placental lipases [1, 8]. To facilitate the requirements of the developing fetus, the concentrations of maternal lipids such as triglycerides and total cholesterol rise over the course of pregnancy [9, 10].

Pregnant women with low cholesterol concentrations have a higher risk for fetal growth restriction (FGR), preterm birth, and small-for-gestational age neonates [11–14]. Women affected by the Smith-Lemli-Opitz syndrome (SLOS), an inherited metabolic disease that results in a decreased cholesterol production, are at a higher risk of giving birth to small-for-gestational age neonates [2, 12]. Low LDL-c has even been proposed as a clinical marker for FGR risk assessment [15]. In contrast, a growing body of evidence from animal and human studies also suggests adverse consequences of increased lipid concentrations in pregnancy. High maternal total cholesterol and triglyceride concentrations are associated with an increased risk of hypertensive disorders of pregnancy, (induced) preterm birth and large for gestational age (LGA) neonates [16–18]. Additionally, triglycerides and remnant cholesterol are associated with higher birth weight and infant weight, as well as with the risk of LGA-related complications [18–20]. This is in line with the fetal over-nutrition hypothesis, which suggests that apart from maternal glucose concentrations, other maternal nutrients also contribute to (excess) fetal growth [21]. Additionally, it is proposed that in case of maternal obesity, there is an increased availability of these nutrients and thereby an increased risk for this over-nutrition [21].

Due to the increase in a sedentary lifestyle and a higher intake of calories, a growing number of women of reproductive age are obese and have abnormally elevated lipid levels [22, 23]. As a consequence of these abnormally elevated lipid levels, more women are at risk for an adverse course and outcome of pregnancy [16, 17]. These adverse outcomes do not only affect health of the offspring in the short term, but also have far reaching effects on the health of the offspring in adulthood [24–26]. Therefore, it is important to identify and mitigate factors that have an adverse effect on embryonic and fetal growth and birth outcomes, both for the long- and short-term health of the offspring.

Until recently, most studies focused on the association between the maternal lipid profile and fetal development in the second and third trimester of pregnancy and birthweight. However, we hypothesize that an effect of maternal lipids on embryonic size in early pregnancy may already be present. This is substantiated by the fact that embryonic size early in pregnancy is strongly associated with fetal size throughout pregnancy, and birth outcomes [27, 28]. Our aim was therefore to investigate the association between the maternal lipid profile in early pregnancy and embryonic size.

## Methods

### Design and study population

**Fig. 1:**
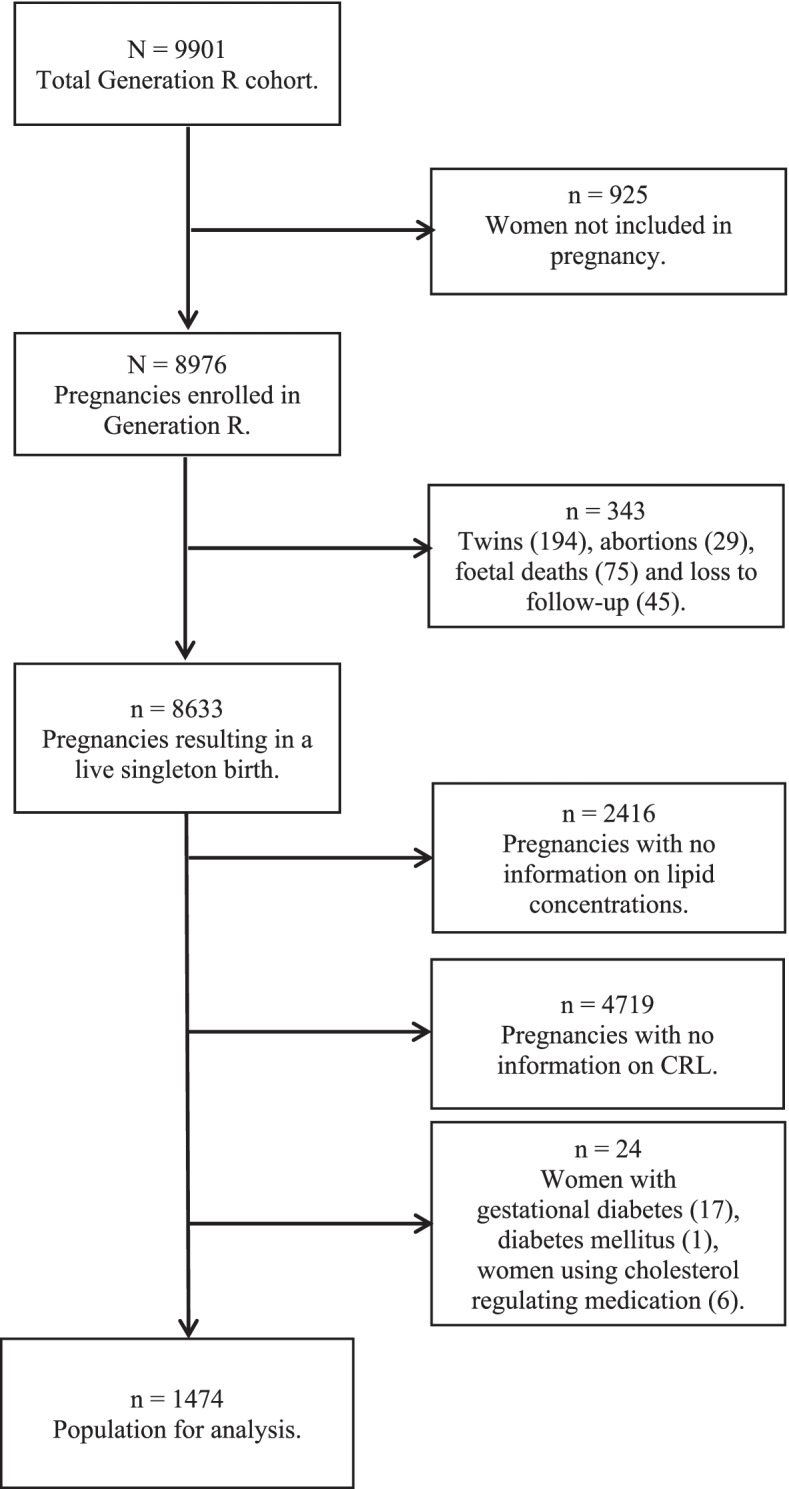
Flowchart of the study population

**Fig. 2:**
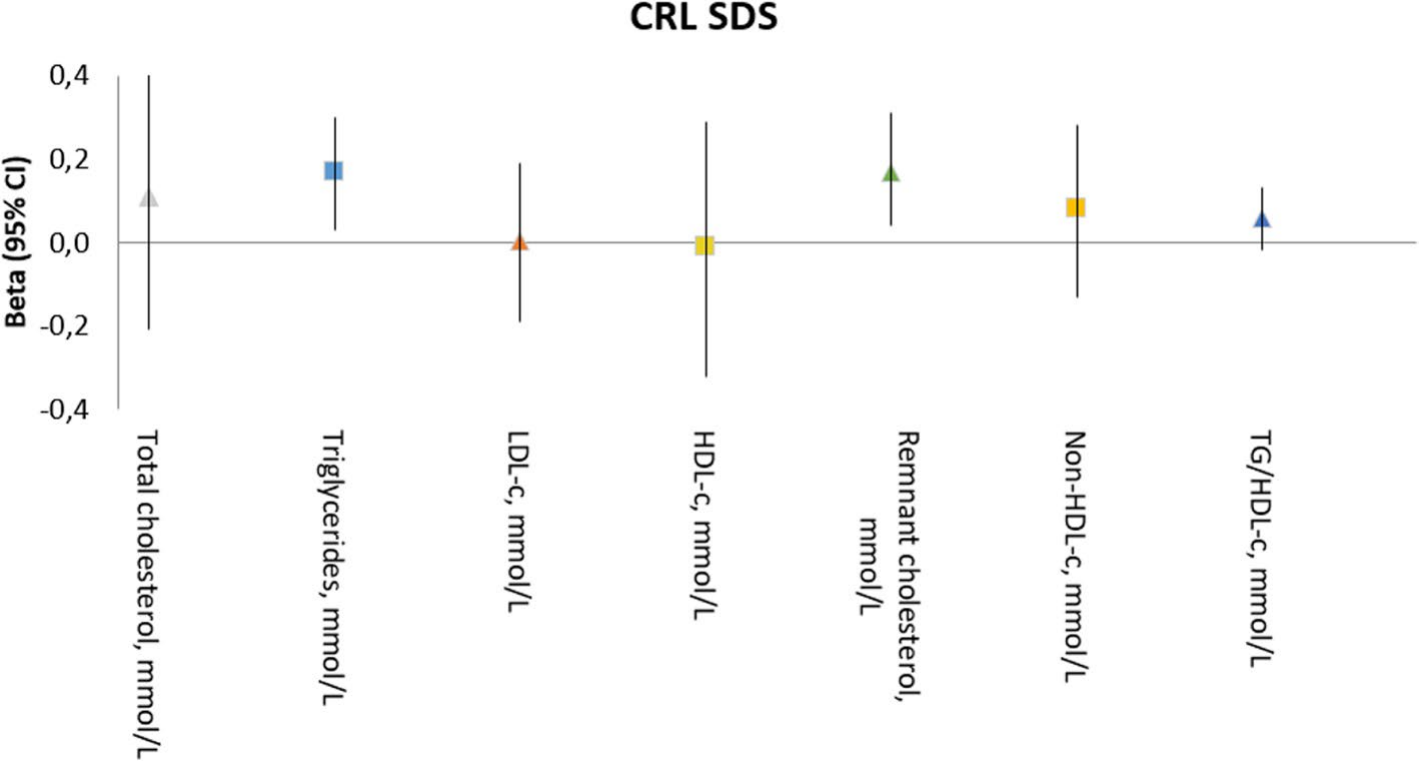
Associations of the maternal lipid profile in early pregnancy with embryonic growth. Abbreviations: CI, confidence interval; LDL-c, low-density lipoprotein cholesterol; HDL-c, high-density lipoprotein cholesterol. Values are linear regression coefficients (95% confidence interval). Adjusted for: maternal age, pre-pregnancy BMI, parity, educational level, ethnicity, smoking, folic acid supplement use and glucose concentrations

This study was embedded in the Generation R Study, a large multi-ethnic population-based prospective cohort study in the city of Rotterdam, the Netherlands [29, 30]. All methods were carried out in accordance with the Declaration of Helsinki. The study protocol has been approved by the Medical Ethics Committee of the Erasmus University Medical Centre (Erasmus MC), Rotterdam (MEC-2007-413). Written informed consent was obtained from all participants. We excluded women that were not pregnant when included in the Generation R study (*n* = 925). Next we excluded twin pregnancies, abortions, fetal deaths and women lost to follow-up (*n* = 343). From the 8633 pregnancies resulting in a live singleton birth, we excluded pregnancies without information on lipid concentrations (*n* = 2416), pregnancies without information on CRL (*n* = 4719), pregnancies complicated by (gestational) diabetes [18] and pregnancies in which cholesterol regulating medication was used [6]. The study population comprised 1474 women with a live born singleton and of whom information was available on lipid measurements and ultrasonic assessment of embryonic size (Figs. 1 and 2).

### Maternal lipid and glucose concentrations in early pregnancy

Non-fasting blood was sampled early in pregnancy (median 12 + 3 weeks of gestation, 90% range [10 + 1–13 + 6 weeks]) by trained research nurses. Details of the processing procedures have been described earlier [30]. After thawing, the total cholesterol (mmol/L) and HDL-c (mmol/L) concentrations were determined using standard laboratory methods. Concentrations of LDL-c were calculated using the Friedewald equation [31]. This calculation is not valid when the triglyceride level is ≥5 mmol/L. In this study population, there are no women with triglycerides above 5 mmol/L. Remnant cholesterol was calculated as the total cholesterol minus LDL-c and minus HDL-c ([total cholesterol – LDL-c] – HDL-c). Non-HDL-c was calculated by subtracting HDL-c from total cholesterol (total-cholesterol – HDL-c). The TG/HDL-ratio was calculated by TG divided by the HDL-c concentration (TG/ HDL-c) (Additional Table 1). Both cholesterol, TG and glucose (mmol/l) were measured with the c702 module on a Cobas 8000 analyzer (Roche, Almere, The Netherlands). Results on maternal lipid levels in this cohort have previously been published [18].

### Embryonic size and birth weight

Embryonic size was assessed by ultrasound examinations using an Aloka model SSD-1700 (Tokyo, Japan) or the ATL-Philips Model HDI 5000 (Seattle, WA, USA). Ultrasound examinations for this study were performed by dedicated ultrasonographers at each prenatal visit to the designated research centers [29]. The crown-rump length (CRL) was measured in a true mid-sagittal plane with the genital tubercle and the spine longitudinally in view, according to standard procedures [27, 32, 33]. Intra-class correlation coefficients for intra-observer and inter-observer reproducibility of crown to rump length measurements were 0.998 and 0.995 [32]. Gestational age (GA) adjusted standard deviation scores (SDS) were constructed for the CRL measurements. Gestational age of the embryo’s was expressed in weeks of gestational age. These scores were based on reference growth curves from the whole study population and represent the equivalent of Z-scores [34]. We obtained information on birth weight from midwifery and obstetric medical records. Gestational-age-adjusted SDS for birth weight were constructed using North European growth standards as the reference growth curve and represent the equivalent of z-scores [34, 35].

### Pregnancy dating

The gestational age is the most important determinant of embryonic and fetal growth. In clinical practice, pregnancy dating is based on the CRL. However, for the purpose of analyses with CRL as the outcome, gestational age should be based on the LMP [36]. In this study, pregnancy dating was thus based on the last known menstrual period in women with a regular menstrual cycle (28 days, range 24–32 days) [27]. The first day of the last menstrual period was derived from the referral letter of the community midwife or hospital [27]. At the ultrasound visit, we checked this date with the mother and obtained additional information on the regularity and duration of the menstrual cycle.

### Covariates

In a consensus meeting (DG, AP, ES, JRvL), we identified confounders for the association between maternal lipid concentrations and embryonic size. This resulted in a Directed Acyclic Graph (DAG) (Supplementary Fig. 1) [37]. The identified confounders were: maternal age (continuous), pre-pregnancy BMI (continuous), parity (nulliparous, multiparous), educational level (no education finished, lower education, middle education, higher education), ethnicity (Dutch and Western, Turkish and Moroccan, African, Asian), smoking (never smoked during pregnancy, smoked until pregnancy was known, continued smoking in pregnancy), folic acid supplement use (started preconceptionally, started in first 10 weeks of pregnancy, no folic acid supplement intake) and glucose concentrations (continuous). Maternal questionnaires at study enrolment provided information on maternal age, pre-pregnancy body mass index, parity, educational level, ethnicity, smoking habits and folic acid supplement use.

### Maternal anthropometrics

We collected information about pre-pregnancy weight by questionnaire, and measured height and weight at enrollment. Questionnaire based weight and measured height were then used to calculate body mass index (BMI)(kg/m^2^). The correlation of pre-pregnancy weight obtained by questionnaire and weight measured at enrollment was high (β = 0.97, *P* < 0.01) [38]. Normal weight was defined as a BMI < 25.0 kg/m^2^ and overweight was defined as a BMI ≥ 25.00 kg/m^2^.

### Statistical analysis

First, baseline characteristics and the distribution of the covariates were determined. We examined potential differences in baseline characteristics between women included and excluded from the analysis. Differences in continuous variables with a normal distribution (mean, SD) were analyzed with Students t-test, and variables with a skewed distribution (median, 90% range) with the Mann-Whitney U test. Categorical variables were analyzed with chi-square tests (Additional Table 2).

Second, multivariate linear regression analyses were performed to study the association between differences in embryonic size for the lower and upper tertiles of the maternal lipid concentrations, compared to the middle tertile. We carried out tests for trends based on multiple linear regression models with the maternal lipid concentrations as a continuous variable. To allow mutual comparison of the lipid measures, we constructed Multiple of the Median (MoM) scores of all lipid measures. This MoM score indicates of how far the individual measure deviates from the median in the study population. The crude model was the univariate analysis of maternal lipid concentrations and embryonic size. In the adjusted model, we additionally corrected for the previously determined confounding factors.

Since lipid changes may not only be the consequence of higher glucoses, but may also cause disturbances of glucose metabolism, there is an interactive effect of the maternal lipid status and maternal glucose status [39]. This changes the direction in the DAG graph, indicating that there may be a confounding or mediating effect of maternal glucose level. We examined whether maternal glucose concentrations mediated the association of maternal lipid concentrations with size growth by adding it to our models (fully adjusted model).

We aimed to investigate the effect of the switch in nutritional source of the embryo, from uterine glands and yolk sac to the placenta, which occurs at around week 12 of gestation. Therefore, sensitivity analyses were performed. Associations between the maternal lipid status and embryonic size were separately investigated in the period of 10 to 12 weeks GA versus 12 to 14 weeks GA, with gestational-age adjusted MoM’s (Additional Table 3). With other sensitivity analyses, we tested the effect of the lowest lipid concentrations by assessing the cases with the lowest 5% of the lipid concentrations (Additional Table 4). Results of all linear regression analyses are presented as SDS with a 95% confidence interval (CI).

The following confounders had missing values: pre-pregnancy BMI (14.3%), parity (0.4%), educational level (4.3%), ethnicity (2.1%), smoking (8.8%), folic acid supplement use (19.3%) and glucose (2.7%). To prevent bias associated with missing data, we used multiple imputations for covariates with missing values. We imputed missing data on the basis of the correlation of missing variables with other participant characteristics, according to the Markov Chain Monte Carlo method [40]. Ten datasets were created and analyzed together. A sensitivity analysis was performed to observe differences in observed and expected values of confounders before and after imputation (Additional Table 5). Lastly, complete case analyses were conducted, in which datasets were used without the imputed missing values (data not shown)**.**

We used IBM Statistical Package of Social Sciences version 25.0 for Windows (SPSS Incl., Chicago, IL, USA) for all statistical analyses. A *p*-value < 0.05 was considered statistically significant.

## Results

Maternal baseline characteristics and first trimester reference ranges (41) for lipid concentrations are presented in Additional Table 6. In the study we included 1474 women. Women were on average 30.8 (±4.6) years of age, 1060 (71.9%) women had a Dutch and Western ethnicity and the median pre-pregnancy BMI was 22.6 kg/m^2^ (90% range 18.9; 29.6) (Table 1). Additional Table 2 shows baseline characteristics of women included and excluded from the analyses. Excluded women were on average younger, less often of Dutch and Western ethnicity, more often lower educated and they more often consumed alcohol in pregnancy.

**Table 1:** Baseline characteristics of the study population

Maternal characteristics ***N*** = 1474		Reference ranges lipid concentrations (41)
Age at intake, years	30.8 (4.6)	
Pre-pregnancy BMI, kg/m^2^	22.6 (18.9; 29.9)	
Overweight women, n (%)	347 (25.4)	
Parity (nulliparous)	877 (59.5)	
Educational level (high)	785 (53.3)	
Ethnicity (Dutch and Western)	1060 (71.9)	
Smoking (continued smoking in pregnancy)	232 (15.7)	
Alcohol (continued alcohol consumption in pregnancy)	650 (44.1)	
Folic acid supplement use (start preconceptional)	756 (51.3)	
Embryonic sex (male)	723 (49.1)	
Glucose, mmol/L	4.41 (0.83)	
Total cholesterol, mmol/L	4.69 (0.81)	3.65–5.44
Triglycerides, mmol/L	1.19 (0.70; 2.24)	0.50–1.80
HDL-c, mmol/L	1.77 (0.34)	1.04–2.02
LDL-c, mmol/L	2.34 (0.67)	1.55–3.96
Remnant cholesterol, mmol/L	0.54 (0.32; 1.01)	–
Non-HDL-c, mmol/L	2.93 (0.77)	–
TG/HDL-c ratio	0.67 (0.34; 1.63)	–

Abbreviations: *HDL-c* high-density lipoprotein cholesterol, *LDL-c* low-density lipoprotein cholesterol, *TG* triglycerides, *BMI* body mass index. Values are means (SD) for continuous variables with a normal distribution, or medians (90% range) for continuous variables with a skewed distribution. Confounders were imputed. Non-imputed values represent valid percentages

The associations between maternal lipid concentrations and CRL are shown in Table 2. In the crude analyses, a larger CRL was observed in women with higher triglyceride concentrations; a significant linear trend was observed (crude model, 0.16 SDS CRL; 95% CI, 0.05; 0.38, per 1 MoM increase).

**Table 2:** Associations of maternal lipid profile in early pregnancy with embryonic growth in whole population, women with normal pre-pregnancy weight and overweight women

Study population (***n*** = 1474)	Crude model			Adjusted model			Fully adjusted model		
	Whole group	Normal weight	Overweight	Whole group	Normal weight	Overweight	Whole group	Normal weight	Overweight
	**β (95% CI)**	**β (95% CI)**	**β (95% CI)**	**β (95% CI)**	**β (95% CI)**	**β (95% CI)**	**β (95% CI)**	**β (95% CI)**	**β (95% CI)**
**Total cholesterol, mmol/L**									
Lowest tertile MoM (< 0.94)	− 0.03 (− 0.15; 0.09)	− 0.08 (− 0.23; 0.07)	0.01 (− 0.26; 0.28)	− 0.07 (− 0.20; 0.07)	− 0.08 (− 0.24; 0.09)	− 0.13 (− 0.44; 0.17)	− 0.06 (− 0.20; 0.07)	− 0.07 (− 0.24; 0.10)	− 0.14 (− 0.46; 0.17)
Second tertile MoM (0.94–1.08)	*Reference*	*Reference*	*Reference*	*Reference*	*Reference*	*Reference*	*Reference*	*Reference*	*Reference*
Highest tertile MoM (> 1.08)	0.02 (− 0.10; 0.14)	− 0.001 (− 0.15; 0.15)	0.12 (− 0.13; 0.37)	− 0.02 (− 0.15; 0.11)	− 0.03 (− 0.20; 0.13)	− 0.02 (− 0.30; 0.26)	− 0.02 (− 0.15; 0.11)	− 0.05 (− 0.22; 0.12)	− 0.04 (− 0.32; 0.25)
Trend analyses MoM	0.09 (− 0.19; 0.37)	0.21 (− 0.15; 0.56)	0.15 (− 0.47; 0.77)	0.13 (− 0.19; 0.44)	0.13 (− 0.26; 0.51)	0.13 (− 0.56; 0.82)	0.11 (− 0.21; 0.43)	− 0.40 (− 1.38; 0.57)	0.07 (− 0.32; 0.47)
**Triglycerides, mmol/L**									
Lowest tertile MoM (< 0.87)	− 0.08 (− 0.20; 0.04)	− 0.12 (− 0.26; 0.03)	0.06 (− 0.24; 0.36)	− 0.08 (− 0.21; 0.06)	− 0.09 (− 0.25; 0.07)	0.03 (− 0.30; 0.36)	− 0.08 (− 0.21; 0.06)	− 0.09 (− 0.25; 0.07)	0.05 (− 0.28; 0.39)
Second tertile MoM (0.87–1.18)	*Reference*	*Reference*	*Reference*	*Reference*	*Reference*	*Reference*	*Reference*	*Reference*	*Reference*
Highest tertile MoM (> 1.18)	0.04 (− 0.08; 0.16)	− 0.01 (− 0.16; 0.15)	0.18 (− 0.07; 0.43)	0.04 (− 0.10; 0.18)	− 0.06 (− 0.23; 0.11)	0.23 (− 0.05; 0.50)	0.06 (− 0.07; 0.20)	− 0.06 (− 0.23; 0.12)	0.28 (− 0.01; 0.56)
Trend analyses MoM	**0.16 (0.05; 0.38)**	0.12 (− 0.03; 0.28)	**0.29 (0.04; 0.53)**	**0.15 (0.01; 0.28)**	0.02 (− 0.15; 0.18)	**0.35 (0.10; 0.61)**	**0.17 (0.03; 0.30)**	0.03 (− 0.15; 0.20)	**0.44 (0.15; 0.72)**
**HDL-c, mmol/L**									
Lowest tertile MoM (< 0.92)	0.04 (− 0.08; 0.16)	0.01 (− 0.14; 0.17)	0.18 (− 0.08; 0.43)	0.08 (− 0.06; 0.22)	0.06 (− 0.11; 0.24)	0.13 (− 0.16; 0.43)	0.10 (− 0.04; 0.23)	0.09 (− 0.09; 0.26)	0.15 (− 0.15; 0.45)
Second tertile MoM (0.92–1.08)	*Reference*	*Reference*	*Reference*	*Reference*	*Reference*	*Reference*	*Reference*	*Reference*	*Reference*
Highest tertile MoM (> 1.08)	0.04 (− 0.08; 0.16)	0.07 (− 0.08; 0.22)	− 0.03 (− 0.31; 0.25)	0.05 (− 0.08; 0.19)	0.06 (− 0.10; 0.22)	− 0.03 (− 0.34; 0.28)	0.05 (− 0.08; 0.18)	0.07 (− 0.09; 0.23)	− 0.05 (− 0.37; 0.27)
Trend analyses MoM	− 0.01 (− 0.26; 0.25)	0.17 (− 0.16; 0.50)	−0.51 (− 1.07; 0.06)	0.03 (− 0.28; 0.32)	0.18 (− 0.20; 0.55)	−0.39 (− 1.03; 0.25)	−0.01 (− 0.32; 0.29)	0.15 (− 0.23; 0.54)	−0.48 (− 1.16; 0.20)
**LDL-c, mmol/L**									
Lowest tertile MoM (< 0.88)	0.04 (−0.08; 0.17)	0.01 (−0.14; 0.16)	−0.09 (− 0.37; 0.19)	−0.01 (− 0.15; 0.13)	−0.06 (− 0.22; 0.11)	−0.05 (− 0.37; 0.27)	−0.01 (− 0.15; 0.13)	−0.04 (− 0.21; 0.13)	−0.05 (− 0.38; 0.28)
Second tertile MoM (0.88–1.13)	*Reference*	*Reference*	*Reference*	*Reference*	*Reference*	*Reference*	*Reference*	*Reference*	*Reference*
Highest tertile MoM (> 1.13)	0.03 (−0.09; 0.15)	0.03 (− 0.12; 0.19)	0.03 (− 0.22; 0.27)	−0.01 (− 0.14; 0.12)	−0.04 (− 0.21; 0.13)	0.02 (− 0.25; 0.30)	−0.01 (− 0.15; 0.12)	−0.05 (− 0.22; 0.12)	0.01 (− 0.27; 0.29)
Trend analyses MoM	−0.02 (− 0.18; 0.15)	0.03 (− 0.18; 0.25)	0.10 (− 0.26; 0.47)	0.01 (− 0.18; 0.20)	0.03 (−0.20; 0.26)	0.02 (− 0.39; 0.43)	0.004 (− 0.19; 0.19)	−0.01 (− 0.24; 0.23)	0.001 (− 0.42; 0.42)
**Remnant cholesterol, mmol/L**									
Lowest tertile MoM (< 0.87)	−0.08 (− 0.20; 0.04)	−0.13 (− 0.28; 0.02)	0.09 (− 0.21; 0.38)	−0.08 (− 0.21; 0.06)	−0.10 (− 0.25; 0.06)	0.06 (− 0.27; 0.39)	−0.08 (− 0.21; 0.06)	−0.10 (− 0.26; 0.06)	0.09 (− 0.25; 0.42)
Second tertile MoM (0.87–1.17)	*Reference*	*Reference*	*Reference*	*Reference*	*Reference*	*Reference*	*Reference*	*Reference*	*Reference*
Highest tertile MoM (> 1.17)	0.05 (−0.08; 0.17)	− 0.04 (− 0.20; 0.12)	0.24 (− 0.01; 0.49)	0.04 (− 0.10; 0.18)	−0.08 (− 0.25; 0.09)	0.27 (− 0.003; 0.55)	0.06 (− 0.08; 0.20)	−0.08 (− 0.25; 0.10)	**0.32 (0.04; 0.61)**
Trend analyses MoM	**0.17 (0.05; 0.29)**	0.13 (− 0.02; 0.29)	**0.29 (0.05; 0.53)**	**0.15 (0.02; 0.29)**	0.02 (− 0.15; 0.19)	**0.35 (0.09; 0.61)**	**0.17 (0.04; 0.31)**	0.03 (− 0.15; 0.20)	**0.44 (0.15; 0.72)**
**Non-HDL-c, mmol/L**									
Lowest tertile MoM (< 0.89)	0.03 (−0.09; 0.15)	0.02 (− 0.13; 0.17)	−0.13 (− 0.41; 0.15)	−0.01 (− 0.14; 0.13)	−0.003 (− 0.17; 0.16)	−0.22 (− 0.54; 0.09)	−0.01 (− 0.15; 0.13)	0.01 (− 0.16; 0.18)	−0.23 (− 0.55; 0.10)
Second tertile MoM (0.89–1.11)	*Reference*	*Reference*	*Reference*	*Reference*	*Reference*	*Reference*	*Reference*	*Reference*	*Reference*
Highest tertile MoM (> 1.11)	0.07 (−0.05; 0.19)	0.09 (− 0.06; 0.25)	0.03 (− 0.22; 0.27)	0.05 (− 0.09; 0.18)	0.03 (− 0.14; 0.20)	−0.03 (− 0.30; 0.24)	0.05 (− 0.09; 0.18)	0.02 (− 0.16; 0.19)	−0.04 (− 0.32; 0.24)
Trend analyses MoM	0.06 (− 0.12; 0.24)	0.09 (− 0.14; 0.32)	0.25 (− 0.15; 0.66)	0.08 (−0.13; 0.28)	0.03 (− 0.21; 0.28)	0.21 (− 0.24; 0.67)	0.08 (− 0.13; 0.28)	0.01 (−0.25; 0.26)	0.21 (− 0.26; 0.68)
**TG/HDL-c ratio**									
Lowest tertile MoM (< 0.83)	−0.07 (−0.19; 0.05)	− 0.01 (− 0.16; 0.13)	−0.15 (− 0.45; 0.15)	−0.04 (− 0.17; 0.09)	0.003 (− 0.15; 0.16)	−0.11 (− 0.44; 0.22)	−0.04 (− 0.17; 0.09)	0.01 (− 0.15; 0.17)	−0.15 (− 0.49; 0.18)
Second tertile MoM (0.83–1.23)	*Reference*	*Reference*	*Reference*	*Reference*	*Reference*	*Reference*	*Reference*	*Reference*	*Reference*
Highest tertile MoM (> 1.23)	0.01 (−0.11; 0.13)	0.02 (− 0.14; 0.18)	0.10 (− 0.16; 0.35)	0.05 (− 0.09; 0.19)	−0.01 (− 0.19; 0.16)	0.26 (− 0.03; 0.54)	0.07 (− 0.07; 0.21)	0.002 (− 0.18; 0.18)	0.28 (−0.01; 0.57)
Trend analyses MoM	0.06 (−0.01; 0.13)	0.03 (− 0.06; 0.12)	**0.15 (0.01; 0.29)**	0.04 (− 0.03; 0.12)	−0.02 (− 0.12; 0.07)	**0.16 (0.01; 0.31)**	0.06 (− 0.02; 0.13)	−0.01 (− 0.11; 0.09)	**0.26 (0.06; 0.45)**

Abbreviations: *CI* confidence interval, *HDL-c* high-density lipoprotein cholesterol, *LDL-c* low-density lipoprotein cholesterol, *MoM* Multiple of the median, *n.a.* not applicable. Values are SDS with the 95% CI and are based on linear regression models. Crude model: univariate regression analysis. Adjusted model: crude model additionally adjusted for maternal age, parity, educational level, ethnicity, smoking and folic acid supplement use. Fully adjusted model: adjusted model additionally adjusted for maternal glucose concentrations. Estimates of MoM trend analyses represent the unit increase in the outcome per 1 multiple of the median increase in lipid, compared to the reference category

In the multivariable analyses, the association remained significant (adjusted model, 0.15 SDS; 95% CI, 0.01; 0.28), also after additionally adjusting for glucose concentrations (fully adjusted model, 0.17 SDS CRL; 95% CI, 0.03; 0.30, per 1 MoM increase). When analyses were performed according to BMI (i.e. normal weight or overweight), the associations only remained in the overweight group (crude model, 0.29 SDS CRL; 95% CI, 0.04; 0.53, per 1 MoM increase. Adjusted model, 0.35 SDS CRL; 95% CI, 0.10; 0.61, per 1 MoM increase and fully adjusted model, 0.44 SDS CRL; 95% CI, 0.15; 0.72, per 1 MoM increase).

The crude analyses between remnant cholesterol and CRL showed significant positive associations (crude model, 0.17 SDS CRL; 95% CI, 0.05; 0.29, per 1 MoM increase). After adjustment for confounders in the multivariable analysis, and the fully adjusted analysis, the significant associations remained (adjusted model, 0.15 SDS CRL; 95% CI, 0.02–0.29, per 1 MoM increase and fully adjusted model, 0.17 SDS CRL; 95% CI, 0.04–0.31,, per 1 MoM increase, respectively). Again, the associations only remained in the overweight group (crude model, 0.29 SDS CRL; 95% CI, 0.05; 0.53, per 1 MoM increase. Adjusted model, 0.35 SDS CRL; 95% CI, 0.09; 0.61, per 1 MoM increase and fully adjusted model, 0.44 SDS CRL; 95% CI, 0.15; 0.72, per 1 MoM increase) (Table 2). Total-cholesterol, HDL-c, LDL-c, non-HDL-c concentrations and the TG/HDL-c ratio in early pregnancy were not associated with CRL. We tested for multicollinearity using the tolerance statistic. As tolerance was > 0.20 for all variables in our models, multicollinearity was unlikely.

Sensitivity analysis demonstrated that the associations between triglycerides and remnant cholesterol and embryonic size attenuated and were no longer significant when the analyses were split for gestational age 10–12 weeks and 12–14 weeks (Additional Table 3). Complete case analysis showed similar results to those presented in Table 2 (data not shown). Also, sensitivity analyses were performed in which we examined the effect of the lowest lipid concentrations within the study population. When investigating the association between the lowest 5% lipid concentrations and embryonic size, no significant associations were observed (fully adjusted model triglycerides, − 0.16 SDS CRL; 95% CI, − 0.38; 0.13, per 1 MoM increase and fully adjusted model remnant cholesterol, − 0.13 SDS CRL; 95% CI, − 0.29; 0.20, per 1 MoM increase, respectively) (Additional Table 4).

## Discussion

### Principal findings

We showed that both maternal triglycerides and remnant cholesterol in early pregnancy are positively associated with embryonic size, especially in overweight women and even after adjustment for glucose concentrations [41].

Lipids such as triglycerides and cholesterol reach the developing embryo or fetus through different mechanisms, which change over the course of pregnancy. In the first 12 weeks of pregnancy, the placenta is developing and not fully functional [42]. In this period, the developing embryo is dependent on the yolk sac and uterine glands for the storage and transport of nutrition [43, 44]. The yolk sac transports maternal lipids into the vitelline vessels that are connected with the circulation of the embryo [45]. Animal studies showed that as the maternal serum lipid concentrations increased, so did the concentrations in the yolk sac, and consequently the secretion by the yolk sac into the embryo [46]. This indicates that the lipid transport to the embryo is dependent on maternal serum lipid concentrations. For triglycerides to pass the yolk sac membrane, they have to be hydrolyzed into free fatty acids by placental lipases [47]. From animal studies it is known that during embryonic growth, approximately 90% of the total energy requirement is derived from yolk lipid fatty acid oxidation [48]. This indicates triglycerides have an important role as energy source in the development of an embryo, supporting our positive association between triglycerides and embryonic growth. In the performed sensitivity analyses, we were not able to verify the increased dependency of the embryo on the maternal lipid concentrations in the first 12 weeks of pregnancy (analyses split for gestational age 10–12 weeks and 12–14 weeks). Neither did we find stronger associations when we investigated the group of women with the lowest 5% lipid concentrations compared to the higher lipid levels in relation to embryonic size. This could be explained by the fact that by stratifying, the groups are smaller, which lowers the statistical power to detect statistically significant differences. However, the observed effect estimates in these sensitivity analyses are very similar from the main analyses.

Since triglycerides and remnant cholesterol only make up a small part of the total cholesterol content, it could explain why we did not find a positive association between total cholesterol concentrations with embryonic growth. Moreover, based on previous studies, it is postulated that only very low maternal total cholesterol levels are related to fetal and newborn size [13, 49]. In a study demonstrating a negative association between total cholesterol levels and newborn birth weight [13], the mean level of total cholesterol was 3.6 mmol/L. By contrast, the mean level in this study population was 4.8 mmol/L. This possibly explains the absence of an association in this study population. Additionally, we did not find an association between HDL-c levels and embryonic size. This is in line with earlier studies examining the association between HDL-c levels and postnatal measures of fetal growth (i.e. LGA), which also did not observe a significant association [50, 51]. Lastly, we did not find an association between LDL-c and embryonic size. Previous epidemiological studies observed reduced serum LDL-c levels in pregnancies complicated by fetal growth restriction [52]. A suggested underlying mechanism for the association between LDL-c levels and impaired fetal growth is an increased lipid oxidation of LDL-c. These modified LDLs are not recognized nor taken up by the LDL receptor, after which the modified LDL accumulates outside the receptor and initiates plaque formation in the maternal spiral arteries of the placenta. This contributes to artery occlusion, a disturbed perfusion of the placenta and has fetal growth restriction as a result [53, 54]. Because the spiral arteries and placenta are not formed yet in the first trimester of pregnancy, we propose (oxidized) LDL-c does not have the same negative effect on embryonic size.

Strikingly, the demonstrated associations between maternal serum lipid concentrations and embryonic size were most prominent in overweight women. Adiposity is associated with metabolic and endocrinologic changes, and they differ depending on different BMI [55]. The findings of a previous study investigating maternal lipid levels and fetal birthweight, suggest that the metabolic lipid pathways affecting fetal growth may be substantially different in overweight women, compared to the normal weight women [56]. We also find associations with embryonic size in this group of overweight women to be more profound, which may be explained by the possible positive association between maternal BMI and embryonic size [57]. Second, this could be explained by the strong association between both obesity and insulin resistance, and insulin resistance and remnant cholesterol [58–60]. Therefore, the effect of higher lipid levels may be stronger if the woman is insulin resistant. However, since the gold standard for the assessment of insulin resistance is the hyperinsulinemic-euglycemic clamp, and this was not utilized, we were not able to verify this in this study, [61]. Though, the analyses were additionally adjusted for maternal glucose levels, which is an indirect measure of insulin resistance. Apart from small changes in effect estimates, we could not substantiate an insulin dependent effect in overweight women. However, again, we did not make use of the gold standard for insulin resistance. The yolk sac is important for nutrient transport to the embryo. Hypothetically, hyperglycemia in early pregnancy injures the development of the yolk sac, which impairs embryonic growth and development. Next, there appears to be a decrease in glucose transport in embryos exposed to a hyperglycemic environment, which may lead to programmed cell death, and therefore impaired growth and development [62]. However, the analyses in which we additionally adjust for maternal glucose concentrations did not reveal a glucose dependent effect on embryonic size. This is in line with both in vivo and in vitro studies, that could not verify this negative association of maternal glucose levels on early embryonic growth [63, 64]. In this study population, there was a relatively small number of women with high levels of glucose or hyperglycemia, which might have decreased the possible interactive effect on the association between the maternal lipids and embryonic size. Therefore, the observed associations may be stronger among higher-risk populations.

### Clinical implications

Our findings demonstrate that the previously established associations between maternal lipids, fetal growth and adverse birth outcomes may already be present during the first trimester of pregnancy [17, 18, 28, 49, 65, 66]. [20].

It is also in line with the Developmental Origins of Health and Disease (DOHaD) theory, which states that adverse influences in early pregnancy have the potential to affect the change of adverse birth outcomes [24]. The finding that specifically both triglycerides and remnant cholesterol are associated with embryonic size are not surprising, as plasma triglycerides and remnant cholesterol are highly correlated [67]. Remnant cholesterol is the cholesterol content of triglyceride-rich lipoproteins. In a clinical setting, triglycerides are even proposed as a surrogate marker of remnant cholesterol [68, 69]. Additionally, our results emphasize the potential of triglycerides and remnant cholesterol as markers for first trimester size.

### Research implications

To unravel the mechanisms of nutrient transport from mother to embryo, and especially lipid transport, more fundamental research is needed. Also changes in nutrient transport due to the switch from yolk sac and uterine glands to the placenta as main nutrient transporter is interesting. Second, due to the small measures of the CRL, the measurement ranges are also small. Lastly, research with repeated CRL measurements would make it possible to investigate embryonic growth patterns.

### Strengths & limitations

To our knowledge, this is the first study which investigates the association between the maternal lipid profile and embryonic size in early pregnancy. One limitation is that maternal blood samples were obtained in a non-fasting state and not on a specific time of the day, while the levels are sensitive towards intake. This could have led to an underestimation of the observed associations, due to non-differential misclassification of high or low lipid levels. Moreover, multiple studies have demonstrated that plasma lipids only change a little in response to food intake [70–77]. Therefore, non-fasting lipids levels can used to evaluate the serum lipid status of pregnant women instead of fasting lipids. As an exception, only fasting blood samples should be considered if non-fasting plasma triglycerides are above 5 mmol/L. [75] However, in our study population, there were no women with non-fasting triglycerides that exceeded 5 mmol/L.

A second limitation, is that embryonic size was measured only once. Therefore, no patterns of embryonic growth could be assessed. Also no information on (changes in) pre-pregnancy lipid concentrations was available. We therefore cannot investigate the effect of preconceptional lipid concentrations on embryonic size. Next, the use of MoM’s in the analyses makes it harder to clinically interpret the associations. However, these MoM’s enable to compare the different lipid concentrations to each other. The effect sizes for the association between triglycerides and remnant cholesterol and embryonic size are comparable.

Moreover, there might be the issue of response bias or self-selection, which is known to happen in cohort studies. Indeed, the median BMI of 22.6 within our study population is within the healthy range and the majority of women did not smoke during pregnancy (73.9%) (Additional Table 1). Indeed, most of the measured maternal lipid concentrations are within the recommended ranges for the first trimester of pregnancy [78]. The selection of a relatively healthy study population did thus not allow to investigate the associations of extreme dyslipidemia. This might imply that effects in the general population with more and severe dyslipidemia may be even larger, and thus has affected the generalizability of our results. Finally, the observational nature of this study does not allow for inference of causality.

## Conclusions

The positive association between maternal lipids and embryonic size in pregnancy, especially in overweight women, emphasizes the importance of healthy maternal nutrition and a healthy weight. we propose maternal serum lipids concentrations, especially triglycerides and remnant cholesterol, may be a marker for early embryonic and fetal growth. Additionally, they are potentially new targets for an early intervention in overweight pregnant women to prevent excess embryonic and fetal growth.

## Supplementary Information

**Additional file 1**

## Data Availability

Reasonable data requests can be made to the secretary of Generation R.

## Abbreviations

TG: Triglycerides
HDL-c: High-density lipoprotein cholesterol
LDL-c: Low-density lipoprotein cholesterol
non-HDL-c: Non-high-density
CRL: Crown-rump length
BMI: Pregnancy body mass index
FGR: Fetal growth restriction
SLOS: Smith-Lemli-Opitz syndrome
LGA: Large for gestational age
Erasmus MC: Erasmus University Medical Centre
LMP: Last menstrual period
GA: Gestational age
SDS: Standard deviation scores
DAG: Directed Acyclic Graph
MoM: Multiple of the Median
CI: Confidence interval
DOHaD: Developmental Origins of Health and Disease

## References

[1] Herrera E, Desoye G (2016). Maternal and fetal lipid metabolism under normal and gestational diabetic conditions. Horm Mol Biol Clin Investig.

[2] Baardman ME, Kerstjens-Frederikse WS, Berger RM, Bakker MK, Hofstra RM, Plosch T (2013). The role of maternal-fetal cholesterol transport in early fetal life: current insights. Biol Reprod.

[3] Nezil FA, Bloom M (1992). Combined influence of cholesterol and synthetic amphiphillic peptides upon bilayer thickness in model membranes. Biophys J.

[4] Li-Beisson Y, Nakamura Y, Harwood J (2016). Lipids: from chemical structures, biosynthesis, and analyses to industrial applications. Subcell Biochem.

[5] Tuckey RC (2005). Progesterone synthesis by the human placenta. Placenta..

[6] Coukos G, Gafvels ME, Wittmaack F, Matsuo H, Strickland DK, Coutifaris C (1994). Potential roles for the low density lipoprotein receptor family of proteins in implantation and placentation. Ann N Y Acad Sci.

[7] Winkel CA, MacDonald PC, Simpson ER (1981). The role of receptor-mediated low-density lipoprotein uptake and degradation in the regulation of progesterone biosynthesis and cholesterol metabolism by human trophoblasts. Placenta Suppl.

[8] Gauster M, Hiden U, Blaschitz A, Frank S, Lang U, Alvino G (2007). Dysregulation of placental endothelial lipase and lipoprotein lipase in intrauterine growth-restricted pregnancies. J Clin Endocrinol Metab.

[9] Grimes SB, Wild R (2000). Effect of pregnancy on lipid metabolism and lipoprotein levels.

[10] Lippi G, Albiero A, Montagnana M, Salvagno GL, Scevarolli S, Franchi M (2007). Lipid and lipoprotein profile in physiological pregnancy. Clin Lab.

[11] Smith DW, Lemli L, Opitz JM (1964). A newly recognized syndrome of multiple congenital anomalies. J Pediatr.

[12] Cooper MK, Wassif CA, Krakowiak PA, Taipale J, Gong R, Kelley RI (2003). A defective response to hedgehog signaling in disorders of cholesterol biosynthesis. Nat Genet.

[13] Edison RJ, Berg K, Remaley A, Kelley R, Rotimi C, Stevenson RE (2007). Adverse birth outcome among mothers with low serum cholesterol. Pediatrics..

[14] Pecks U, Brieger M, Schiessl B, Bauerschlag DO, Piroth D, Bruno B (2012). Maternal and fetal cord blood lipids in intrauterine growth restriction. J Perinat Med.

[15] Sattar N, Greer IA, Galloway PJ, Packard CJ, Shepherd J, Kelly T (1999). Lipid and lipoprotein concentrations in pregnancies complicated by intrauterine growth restriction. J Clin Endocrinol Metab.

[16] Catov JM, Bodnar LM, Kip KE, Hubel C, Ness RB, Harger G (2007). Early pregnancy lipid concentrations and spontaneous preterm birth. Am J Obstet Gynecol.

[17] Vrijkotte TG, Krukziener N, Hutten BA, Vollebregt KC, van Eijsden M, Twickler MB (2012). Maternal lipid profile during early pregnancy and pregnancy complications and outcomes: the ABCD study. J Clin Endocrinol Metab.

[18] Adank MC, Benschop L, Kors AW, Peterbroers KR, Smak Gregoor AM, Mulder MT (2020). Maternal lipid profile in early pregnancy is associated with foetal growth and the risk of a child born large-for-gestational age: a population-based prospective cohort study : maternal lipid profile in early pregnancy and foetal growth. BMC Med.

[19] Harmon KA, Gerard L, Jensen DR, Kealey EH, Hernandez TL, Reece MS (2011). Continuous glucose profiles in obese and normal-weight pregnant women on a controlled diet: metabolic determinants of fetal growth. Diabetes Care.

[20] Barbour LA, Farabi SS, Friedman JE, Hirsch NM, Reece MS, Van Pelt RE (2018). Postprandial triglycerides predict newborn fat more strongly than glucose in women with obesity in early pregnancy. Obesity (Silver Spring).

[21] Barbour LA (2014). Changing perspectives in pre-existing diabetes and obesity in pregnancy: maternal and infant short- and long-term outcomes. Curr Opin Endocrinol Diabetes Obes.

[22] Chooi YC, Ding C, Magkos F (2019). The epidemiology of obesity. Metabolism..

[23] Klop B, Elte JWF, Cabezas MC (2013). Dyslipidemia in obesity: mechanisms and potential targets. Nutrients..

[24] Barker DJ (2007). The origins of the developmental origins theory. J Intern Med.

[25] Barker DJ, Osmond C (1986). Diet and coronary heart disease in England and Wales during and after the second world war. J Epidemiol Community Health.

[26] Barker DJ, Gluckman PD, Godfrey KM, Harding JE, Owens JA, Robinson JS (1993). Fetal nutrition and cardiovascular disease in adult life. Lancet..

[27] Mook-Kanamori DO, Steegers EA, Eilers PH, Raat H, Hofman A, Jaddoe VW (2010). Risk factors and outcomes associated with first-trimester fetal growth restriction. JAMA..

[28] van Uitert EM, Exalto N, Burton GJ, Willemsen SP, Koning AH, Eilers PH (2013). Human embryonic growth trajectories and associations with fetal growth and birthweight. Hum Reprod.

[29] Jaddoe VW, Mackenbach JP, Moll HA, Steegers EA, Tiemeier H, Verhulst FC (2006). The generation R study: design and cohort profile. Eur J Epidemiol.

[30] Kruithof CJ, Kooijman MN, van Duijn CM, Franco OH, de Jongste JC, Klaver CC (2014). The generation R study: biobank update 2015. Eur J Epidemiol.

[31] Friedewald WT, Levy RI, Fredrickson DS (1972). Estimation of the concentration of low-density lipoprotein cholesterol in plasma, without use of the preparative ultracentrifuge. Clin Chem.

[32] Verburg BO, Mulder PG, Hofman A, Jaddoe VW, Witteman JC, Steegers EA (2008). Intra- and interobserver reproducibility study of early fetal growth parameters. Prenat Diagn.

[33] Robinson HP, Fleming JE (1975). A critical evaluation of sonar "crown-rump length" measurements. Br J Obstet Gynaecol.

[34] Verburg BO, Steegers EA, De Ridder M, Snijders RJ, Smith E, Hofman A (2008). New charts for ultrasound dating of pregnancy and assessment of fetal growth: longitudinal data from a population-based cohort study. Ultrasound Obstet Gynecol.

[35] Niklasson A, Ericson A, Fryer JG, Karlberg J, Lawrence C, Karlberg P (1991). An update of the Swedish reference standards for weight, length and head circumference at birth for given gestational age (1977-1981). Acta Paediatr Scand.

[36] Slama R, Khoshnood B, Kaminski M (2008). How to control for gestational age in studies involving environmental effects on fetal growth. Environ Health Perspect.

[37] Textor J, van der Zander B, Gilthorpe MS, Liskiewicz M, Ellison GT (2016). Robust causal inference using directed acyclic graphs: the R package 'dagitty'. Int J Epidemiol.

[38] Gaillard R, Durmus B, Hofman A, Mackenbach JP, Steegers EA, Jaddoe VW (2013). Risk factors and outcomes of maternal obesity and excessive weight gain during pregnancy. Obesity (Silver Spring).

[39] Parhofer KG (2015). Interaction between glucose and lipid metabolism: more than diabetic dyslipidemia. Diabetes Metab J.

[40] Sterne JA, White IR, Carlin JB, Spratt M, Royston P, Kenward MG (2009). Multiple imputation for missing data in epidemiological and clinical research: potential and pitfalls. BMJ..

[41] Wahab RJ, Scholing JM, Gaillard R (2021). Maternal early pregnancy dietary glycemic index and load, fetal growth, and the risk of adverse birth outcomes. Eur J Nutr..

[42] Burton GJ, Fowden AL (2015). The placenta: a multifaceted, transient organ. Philos Trans R Soc Lond Ser B Biol Sci.

[43] Burton GJ, Jauniaux E, Charnock-Jones DS (2007). Human early placental development: potential roles of the endometrial glands. Placenta..

[44] Burton GJ, Watson AL, Hempstock J, Skepper JN, Jauniaux E (2002). Uterine glands provide histiotrophic nutrition for the human fetus during the first trimester of pregnancy. J Clin Endocrinol Metab.

[45] Lanford RE, Bronson DL, Estlack LE, Wians FH (1991). Plasma protein and apolipoprotein synthesis by human yolk sac carcinoma cells in vitro. In Vitro Cell Dev Biol.

[46] McConihay JA, Horn PS, Woollett LA (2001). Effect of maternal hypercholesterolemia on fetal sterol metabolism in the Golden Syrian hamster. J Lipid Res.

[47] Powell KA, Deans EA, Speake BK (2004). Fatty acid esterification in the yolk sac membrane of the avian embryo. J Comp Physiol B.

[48] Noble RC, Cocchi M (1990). Lipid metabolism and the neonatal chicken. Prog Lipid Res.

[49] Vrijkotte TG, Algera SJ, Brouwer IA, van Eijsden M, Twickler MB (2011). Maternal triglyceride levels during early pregnancy are associated with birth weight and postnatal growth. J Pediatr.

[50] Jin WY, Lin SL, Hou RL, Chen XY, Han T, Jin Y (2016). Associations between maternal lipid profile and pregnancy complications and perinatal outcomes: a population-based study from China. BMC Pregnancy Childbirth.

[51] Emet T, Ustüner I, Güven SG, Balık G, Ural UM, Tekin YB (2013). Plasma lipids and lipoproteins during pregnancy and related pregnancy outcomes. Arch Gynecol Obstet.

[52] Pecks U, Caspers R, Schiessl B, Bauerschlag D, Piroth D, Maass N (2012). The evaluation of the oxidative state of low-density lipoproteins in intrauterine growth restriction and preeclampsia. Hypertens Pregnancy.

[53] Pecks U, Rath W, Caspers R, Sosnowsky K, Ziems B, Thiesen HJ (2013). Oxidatively modified LDL particles in the human placenta in early and late onset intrauterine growth restriction. Placenta..

[54] Oxidized LDL (2010). Diversity, patterns of recognition, and pathophysiology. Antioxid Redox Signal.

[55] Flier JS (2004). Obesity wars: molecular progress confronts an expanding epidemic. Cell..

[56] Misra VK, Trudeau S, Perni U (2011). Maternal serum lipids during pregnancy and infant birth weight: the influence of prepregnancy BMI. Obesity (Silver Spring).

[57] Thagaard IN, Krebs L, Holm JC, Christiansen M, Møller H, Lange T (2018). The effect of obesity on early fetal growth and pregnancy duration: a cohort study. J Matern Fetal Neonatal Med.

[58] Zimmet P, Alberti KG, Shaw J (2001). Global and societal implications of the diabetes epidemic. Nature..

[59] Ohnishi H, Saitoh S, Takagi S, Ohata J, Isobe T, Kikuchi Y (2002). Relationship between insulin-resistance and remnant-like particle cholesterol. Atherosclerosis..

[60] Schaefer EJ, McNamara JR, Shah PK, Nakajima K, Cupples LA, Ordovas JM (2002). Elevated remnant-like particle cholesterol and triglyceride levels in diabetic men and women in the Framingham offspring study. Diabetes Care.

[61] DeFronzo RA, Tobin JD, Andres R (1979). Glucose clamp technique: a method for quantifying insulin secretion and resistance. Am J Phys.

[62] Doblado M, Moley KH (2007). Glucose metabolism in pregnancy and embryogenesis. Curr Opin Endocrinol Diabetes Obes..

[63] Geurtsen ML, van Soest EEL, Voerman E, Steegers EAP, Jaddoe VWV, Gaillard R (2019). High maternal early-pregnancy blood glucose levels are associated with altered fetal growth and increased risk of adverse birth outcomes. Diabetologia..

[64] Ellington SK (1987). In vitro analysis of glucose metabolism and embryonic growth in postimplantation rat embryos. Development..

[65] Gaillard R, Steegers EA, de Jongste JC, Hofman A, Jaddoe VW (2014). Tracking of fetal growth characteristics during different trimesters and the risks of adverse birth outcomes. Int J Epidemiol.

[66] Geraghty AA, Alberdi G, O'Sullivan EJ, O'Brien EC, Crosbie B, Twomey PJ (2016). Maternal blood lipid profile during pregnancy and associations with child adiposity: findings from the ROLO study. PLoS One.

[67] Varbo A, Benn M, Tybjærg-Hansen A, Jørgensen AB, Frikke-Schmidt R, Nordestgaard BG (2013). Remnant cholesterol as a causal risk factor for ischemic heart disease. J Am Coll Cardiol.

[68] Varbo A, Nordestgaard BG (2017). Remnant lipoproteins. Curr Opin Lipidol.

[69] Nordestgaard BG, Varbo A (2014). Triglycerides and cardiovascular disease. Lancet..

[70] Langsted A, Freiberg JJ, Nordestgaard BG (2008). Fasting and nonfasting lipid levels: influence of normal food intake on lipids, lipoproteins, apolipoproteins, and cardiovascular risk prediction. Circulation..

[71] Langsted A, Nordestgaard BG (2011). Nonfasting lipids, lipoproteins, and apolipoproteins in individuals with and without diabetes: 58 434 individuals from the Copenhagen general population study. Clin Chem.

[72] Mora S, Rifai N, Buring JE, Ridker PM (2008). Fasting compared with nonfasting lipids and apolipoproteins for predicting incident cardiovascular events. Circulation..

[73] Sidhu D, Naugler C (2012). Fasting time and lipid levels in a community-based population: a cross-sectional study. Arch Intern Med.

[74] Steiner MJ, Skinner AC, Perrin EM (2011). Fasting might not be necessary before lipid screening: a nationally representative cross-sectional study. Pediatrics..

[75] Nordestgaard BG, Langsted A, Mora S, Kolovou G, Baum H, Bruckert E (2016). Fasting is not routinely required for determination of a lipid profile: clinical and laboratory implications including flagging at desirable concentration cut-points-a joint consensus statement from the European atherosclerosis society and European Federation of Clinical Chemistry and Laboratory Medicine. Eur Heart J.

[76] Langsted A, Nordestgaard BG (2019). Nonfasting versus fasting lipid profile for cardiovascular risk prediction. Pathology..

[77] Li Y, He J, Zeng X, Zhao S, Wang X, Yuan H (2019). Non-fasting lipids detection and their significance in pregnant women. Lipids Health Dis.

[78] Abbassi-Ghanavati M, Greer LG, Cunningham FG (2009). Pregnancy and laboratory studies: a reference table for clinicians. Obstet Gynecol.

