# Supplementary material for: Maternal lipid profile in pregnancy and embryonic size: a population-based prospective cohort study
Source: BMC Pregnancy Childbirth. 2022 Apr 18;22:333. doi: 10.1186/s12884-022-04647-6 (PMC9016996; doi:10.1186/s12884-022-04647-6)
Supplement: Supplementary file 1 — Additional file 1 [file 12884_2022_4647_MOESM1_ESM.docx]

**Supplementary material**

**Additional Table 1.** Calculation of different lipid measurements.

| **Total cholesterol** | **=** | Measured directly |
| --- | --- | --- |
| **Triglycerides** | **=** | Measured directly |
| **High density lipoprotein cholesterol (HDL-c)** | **=** | Measured directly |
| **Low density lipoprotein cholesterol (LDL-c)** | = | Friedewald equation: Total cholesterol - (triglyceride / 5) – HDL-c |
| **Remnant cholesterol** | = | Total cholesterol - LDL-c - HDL-c |
| **Non-high density lipoprotein cholesterol** | = | Total cholesterol – HDL-c |
| **Triglycerides/high density lipoprotein cholesterol-ratio (TG/HDL-c)** | = | Triglycerides/ HDL-c |

Abbreviations: HDL-c: high-density lipoprotein cholesterol, LDL-c: low-density lipoprotein cholesterol.

**Additional Table 2.** Baseline characteristics of women included and excluded in this study (Total N=9901).

| **Maternal characteristics** | **Included**  **n = 1474** | **Excluded**  **n = 8427** | **p-value** |
| --- | --- | --- | --- |
| Age at intake, years | 30.8 (4.6) | 29.8 (5.5) | **<0.001** |
| Pre-pregnancy BMI, kg/m^2^ | 22.6 (18.9 ; 29.9) | 22.7 (18.6 ; 32.9) | 0.01 |
| Parity (nulliparous) | 877 (59.5) | 4508 (53.5) | **<0.001** |
| Educational level (high) | 785 (53.3) | 3230 (38.3) | **<0.001** |
| Ethnicity (Dutch and Western) | 1060 (71.9) | 4777 (56.7) | **<0.001** |
| Smoking (continued smoking in pregnancy) | 232 (15.7) | 1582 (18.8) | **0.001** |
| Alcohol (continued alcohol consumption in pregnancy) | 650 (44.1) | 2954 (35.1) | **<0.001** |
| Folic acid supplement use (start preconceptional) | 756 (51.3) | 3064 (36.4) | **<0.001** |
| Embryonic gender, male | 723 (49.1) | 4215 (51.0) | 0.22 |
| Glucose, mmol/L | 4.41 (0.83) | 4.40 (0.86) | 0.85 |
| Total cholesterol, mmol/L | 4.69 (0.81) | 4.84 (0.87) | **<0.001** |
| Triglycerides, mmol/L | 1.19 (0.70 ; 2.24) | 1.29 (0.72 ; 2.43) | **<0.001** |
| HDL-c, mmol/L | 1.77 (0.34) | 1.76 (0.35) | 0.61 |
| LDL-c, mmol/L | 2.34 (0.67) | 2.44 (0.73) | **<0.001** |
| Remnant cholesterol, mmol/L | 0.54 (0.32 ; 1.01) | 0.59 (0.33 ; 1.10) | **<0.001** |
| Non-HDL-c, mmol/L | 2.93 (0.77) | 3.08 (0.84) | **<0.001** |
| TG/HDL-c ratio | 0.67 (0.34 ; 1.63) | 0.72 (0.36 ; 1.81) | **<0.001** |

Abbreviations: HDL-c, high-density lipoprotein cholesterol; LDL-c, low-density lipoprotein cholesterol; TG, triglycerides; BMI, body mass index. Values are percentages for categorical variables, means (SD) for continuous variables with a normal distribution, or medians (90% range) for continuous variables with a skewed distribution.

**Additional Table 3.** Associations of maternal lipid profile in early pregnancy with crown-rump length, split by period 1 (10 to 12 weeks GA) and 2 (12 to 14 weeks GA).

| **Study population**  **n = 1474** | **Week 10 – 12 GA**  **n = 539** | | | | | | **Week 12 – 14 GA**  **n = 935** | | | | | | |
| --- | --- | --- | --- | --- | --- | --- | --- | --- | --- | --- | --- | --- | --- |
|  | **Crude**  **model** |  | **Adjusted**  **model** |  | **Fully adjusted model** |  | **Crude**  **model** |  | **Adjusted model** |  | **Fully adjusted model** |  |  |
|  | **β (95% CI)** | **p-value** | **β (95% CI)** | **p-value** | **β (95% CI)** | **p-value** | **β (95% CI)** | **p-value** | **β (95% CI)** | **p-value** | **β (95% CI)** | **p-value** |  |
| **Total cholesterol, mmol/L** |  |  |  |  |  |  |  |  |  |  |  |  |  |
| Trend analyses MoM | 0.06  (-0.44 ; 0.55) | 0.83 | 0.34  (-0.21 ; 0.88) | 0.22 | 0.32  (-0.23 ; 0.87) | 0.25 | 0.12  (-0.23 ; 0.47) | 0.50 | -0.01  (-0.41 ; 0.38) | 0.95 | -0.02  (-0.42 ; 0.37 ) | 0.91 |  |
| **Triglycerides, mmol/L** |  |  |  |  |  |  |  |  |  |  |  |  |  |
| Trend analyses MoM | 0.19  (-0.02 ; 0.41) | 0.08 | 0.22  (-0.01 ; 0.45) | 0.06 | 0.23  (-0.01 ; 0.46) | 0.06 | 0.14  (-0.01 ; 0.29) | 0.06 | 0.11  (-0.06 ; 0.28) | 0.20 | 0.15  (-0.02 ; 0.32) | 0.09 |  |
| **HDL-c, mmol/L** |  |  |  |  |  |  |  |  |  |  |  |  |  |
| Trend analyses MoM | -0.01  (-0.46 ; 0.44) | 0.96 | 0.04  (-0.48 ; 0.56) | 0.89 | 0.01  (-0.52 ; 0.53) | 0.98 | -0.004  (-0.32 ; 0.31) | 0.98 | -0.01  (-0.38 ; 0.35) | 0.94 | -0.06  (-0.43 ; 0.31) | 0.75 |  |
| **LDL-c, mmol/L** |  |  |  |  |  |  |  |  |  |  |  |  |  |
| Trend analyses MoM | -0.04  (-0.34 ; 0.26) | 0.79 | 0.14  (-0.19 ; 0.47) | 0.40 | 0.13  (-0.20 ; 0.46) | 0.43 | 0.01  (-0.20 ; 0.21) | 0.95 | -0.07  (-0.30 ; 0.17) | 0.59 | -0.07  (-0.30 ; 0.16) | 0.55 |  |
| **Remnant cholesterol, mmol/L** |  |  |  |  |  |  |  |  |  |  |  |  |  |
| Trend analyses MoM | 0.19  (-0.03 ; 0.40) | 0.08 | 0.22  (-0.02 ; 0.45) | 0.07 | 0.22  (-0.01 ; 0.46) | 0.06 | **0.15**  **(0.003 ; 0.30)** | **0.046** | 0.12  (-0.05 ; 0.29) | 0.18 | 0.15  (-0.02 ; 0.32) | 0.08 |  |
| **Non-HDL-c, mmol/L** |  |  |  |  |  |  |  |  |  |  |  |  |  |
| Trend analyses MoM | 0.04  (-0.28 ; 0.37) | 0.79 | 0.22  (-0.13 ; 0.57) | 0.21 | 0.22  (-0.13 ; 0.57) | 0.23 | 0.07  (-0.15 ; 0.30) | 0.52 | -0.01  (-0.26 ; 0.24) | 0.94 | -0.002  (-0.26 ; 0.25) | 0.99 |  |
| **TG/HDL-c ratio** |  |  |  |  |  |  |  |  |  |  |  |  |  |
| Trend analyses MoM | 0.07  (-0.05 ; 0.19) | 0.25 | 0.06  (-0.07 ; 0.19) | 0.37 | 0.06  (-0.07 ; 0.20) | 0.34 | 0.05  (-0.03 ; 0.13) | 0.22 | 0.04  (-0.05 ; 0.14) | 0.39 | 0.06  (-0.04 ; 0.16) | 0.22 |  |

Abbreviations: GA: gestational age, CI: confidence interval, HDL-c: high-density lipoprotein cholesterol, LDL-c: low-density lipoprotein cholesterol, MoM: Multiple of the median. Values are regression coefficients with the 95% CI and are based on linear regression models. Crude model: univariate regression analysis. Adjusted model: basic model additionally adjusted for maternal age, pre-pregnancy BMI, parity, educational level, ethnicity, smoking and folic acid supplement use. Fully adjusted model: adjusted model additionally adjusted for maternal glucose concentrations.

**Additional Table 4.** Associations of low lipid concentrations (<5^th^ percentile) with embryonic growth.

| **Study population (n=1474)** | **>5th percentile** | **<5th percentile** |  |
| --- | --- | --- | --- |
|  |  | **β (95% CI)** | **p-value** |
| **Total cholesterol** |  | **n=73, <3,45 mmol/L** |  |
| Crude model | *Reference* | 0.06 (-0.17 ; 0.29) | 0.53 |
| Adjusted model | *Reference* | 0.07 (-0.19 ; 0.33) | 0.59 |
| Fully adjusted model | *Reference* | 0.08 (-0.18 ; 0.35) | 0.62 |
| **Triglycerides** |  | **n=67, <0,70 mmol/L** |  |
| Crude model | *Reference* | -0.18 (-0.41 ; 0.06) | 0.15 |
| Adjusted model | *Reference* | -0.12 (-0.37 ; 0.14) | 0.36 |
| Fully adjusted model | *Reference* | -0.13 (-0.38 ; 0.13) | 0.32 |
| **HDL-c** |  | **n=76, <1,20 mmol/L** |  |
| Crude model | *Reference* | 0.13 (-0.09 ; 0.35) | 0.25 |
| Adjusted model | *Reference* | 0.02 (-0.25 ; 0.29) | 0.90 |
| Fully adjusted model | *Reference* | 0.03 (-0.24 ; 0.30) | 0.80 |
| **LDL-c** |  | **n=73, <1,34 mmol/L** |  |
| Crude model | *Reference* | 0.03 (-0.20 ; 0.26) | 0.80 |
| Adjusted model | *Reference* | -0.01 (-0.27 ; 0.25) | 0.94 |
| Fully adjusted model | *Reference* | -0.01 (-0.26 ; 0.25) | 0.96 |
| **Remnant cholesterol** |  | **n=67, < 0,32 mmol/L)** |  |
| Crude model | *Reference* | -0.18 (-0.41 ; 0.06) | 0.14 |
| Adjusted model | *Reference* | -0.12 (-0.37 ; 0.14) | 0.36 |
| Fully adjusted model | *Reference* | -0.13 (-0.38 ; 0.13) | 0.33 |
| **Non-HDL-c** |  | **n=73, <1,77 mmol/L** |  |
| Crude model | *Reference* | 0.07 (-0.16 ; 0.30) | 0.54 |
| Adjusted model | *Reference* | 0.05 (-0.20 ; 0.30) | 0.72 |
| Fully adjusted model | *Reference* | 0.05 (-0.20 ; 0.30) | 0.71 |
| **TG/HDL-c ratio** |  | **n=73, <0,34** |  |
| Crude model | *Reference* | -0.06 (-0.28 ; 0.17) | 0.63 |
| Adjusted model | *Reference* | -0.02 (-0.27 ; 0.22) | 0.85 |
| Fully adjusted model | *Reference* | -0.05 (-0.29 ; 0.20) | 0.71 |

Results from linear regression analysis with MoM’s of lipid concentrations. Abbreviations: CI: confidence interval, HDL-c: high-density lipoprotein cholesterol, LDL-c: low-density lipoprotein cholesterol, MoM: Multiple of the median. Values are regression coefficients with the 95% CI and are based on linear regression models. Crude model: univariate regression analysis. Adjusted model: basic model additionally adjusted for maternal age, pre-pregnancy BMI, parity, educational level, ethnicity, smoking and folic acid supplement use. Fully adjusted model: adjusted model additionally adjusted for maternal glucose concentrations. Estimates of MoM trend analyses represent the unit increase in the outcome per 1 multiple of the median increase in lipid, compared to the reference category.

**Additional Table 5.** Observed and expected values for confounders.

| **Maternal characteristics**  **n=1474** |  | **Expected** | **Observed** |
| --- | --- | --- | --- |
| Age at intake, years | *n=1474* | 30.8 (4.6) | 30.8 (4.6) |
| Pre-pregnancy BMI, kg/m^2^ | *n=1263* | 22.6 (18.9 ; 29.9) | 22.6 (18.9 ; 29.9) |
| Parity (nulliparous) | *n=1468* | 874 (59.5) | 877 (59.5) |
| Educational level (high) | *n=1411* | 772 (54.7) | 785 (53.3) |
| Ethnicity (Dutch and Western) | *n=1443* | 1048 (72.6) | 1060 (71.9) |
| Smoking (continued smoking in pregnancy) | *n=1344* | 210 (14.2) | 232 (15.7) |
| Folic acid supplement use (start preconceptional) | *n=1190* | 639 (53.7) | 759 (51.3) |
| Glucose, mmol/L | *n=1434* | 4.41 (0.83) | 4.41 (0.83) |

Abbreviations: Values are means (SD) for continuous variables with a normal distribution, or medians (90% range) for continuous variables with a skewed distribution.

**Additional Table 6.** Reference ranges of lipid concentrations.

| **Maternal characteristics**  **N=1474** | **Reference ranges**  **lipid concentrations (41)** |
| --- | --- |
| Total cholesterol, mmol/L | 3.65 – 5.44 |
| Triglycerides, mmol/L | 0.50 – 1.80 |
| HDL-c, mmol/L | 1.04 – 2.02 |
| LDL-c, mmol/L | 1.55 – 3.96 |
| Remnant cholesterol, mmol/L | - |
| Non-HDL-c, mmol/L | - |
| TG/HDL-c ratio | - |

Abbreviations: HDL-c, high-density lipoprotein cholesterol; LDL-c, low-density lipoprotein cholesterol; TG, triglycerides.


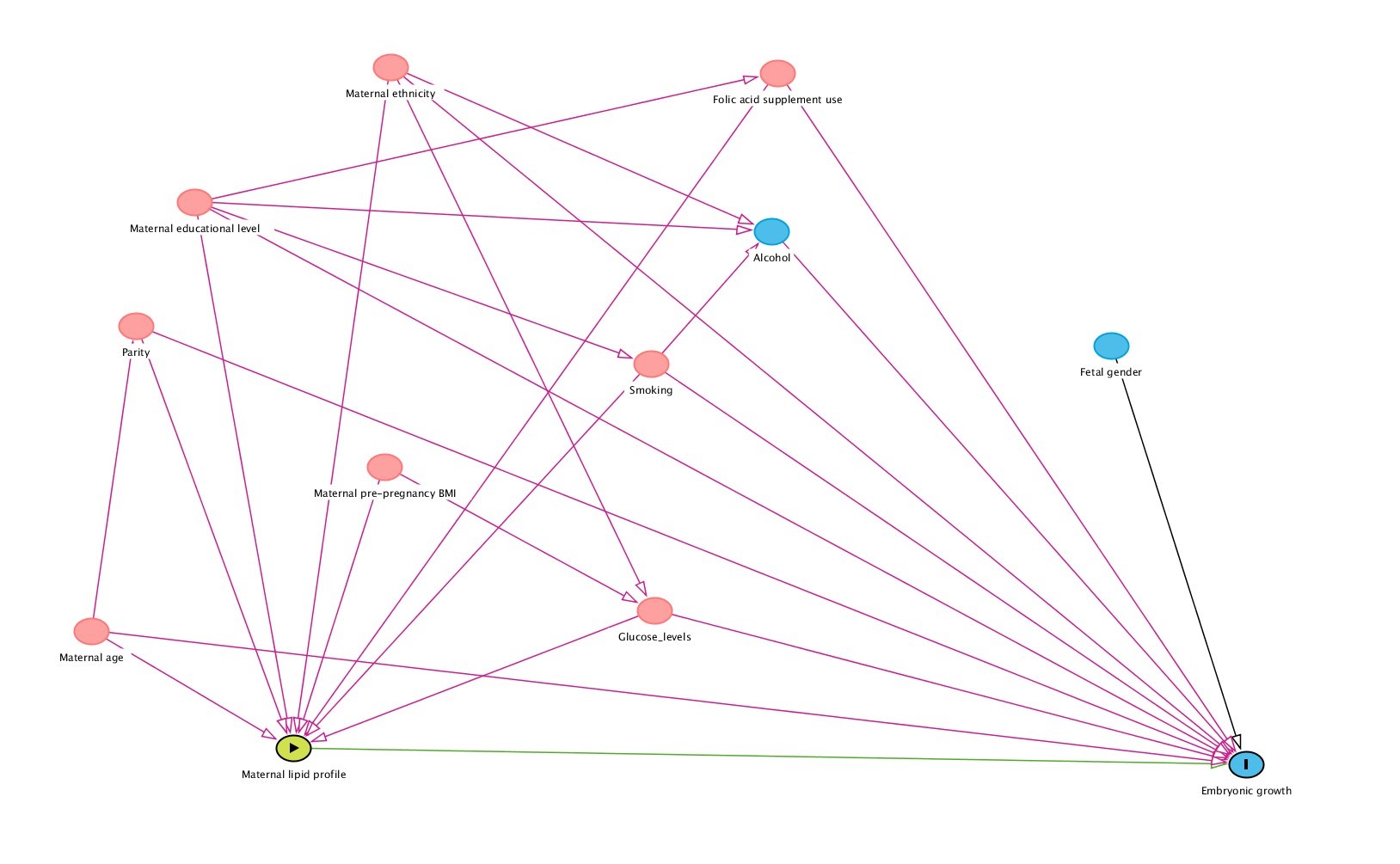
 **Additional Figure 1.** Directed Acyclic Graph (DAG) representing the pathways between the maternal lipid profile and embryonic growth. Abbreviations: BMI, body mass index; CRL, crown-rump length. These confounders (red) were included in the adjusted regression models.
